# Language-Guided Dual-Mode Policy for Dual-Arm Manipulation

**DOI:** 10.3390/s26154883

**Published:** 2026-08-03

**Authors:** Jianghao Sun, Pengjun Mao, Yu Wang, Wenguang Guo

**Affiliations:** School of Mechanical and Electrical Engineering, Henan University of Science and Technology, Luoyang 471000, China; jianghao@stu.haust.edu.cn (J.S.); yu_wang@haust.edu.cn (Y.W.); 240320010097@stu.haust.edu.cn (W.G.)

**Keywords:** dual-arm robot, language-guided, dual-mode, FiLM

## Abstract

Dual-arm robot manipulation tasks are highly complex, and different tasks can be categorized as dual-arm synchronous coordination or asynchronous sequential execution. Existing action-generative policies adopt a fully parameter-sharing single-network architecture in multi-task learning, overlooking the differences between these two task types in terms of temporal dependencies and action distributions, which leads to cross-mode interference. To address this limitation, we propose LGDM (Language-Guided Dual-Mode Policy), a language-guided dual-mode policy framework. Under shared language embeddings, it parallelly constructs dual-conditional branches for language routing and visual perception. The execution layer selects independent synchronous or asynchronous policy branches according to routing signals and outputs actions by combining them with perceptual features, thereby explicitly decoupling the two motion modes. Specifically, the model organizes three functional modules around language embeddings as a hub: (1) a language mode routing layer that predicts motion modes from temporal information in semantics and provides mode-selection signals to the execution layer; (2) a vision perception layer that injects semantic information into the visual backbone via FiLM, enabling task-aware dynamic feature modulation; and (3) a dual-mode execution layer that builds independent synchronous and asynchronous policy branches, selects the corresponding branch according to routing, and generates actions with the modulated features. On the RoboTwin 2 dual-arm manipulation benchmark comprising six synchronous and asynchronous tasks, LGDM outperforms existing baselines, achieving an absolute improvement of 14.5 percentage points over RDT, and maintains robust execution performance under unseen temporal-coordination instruction variants at inference time.

## 1. Introduction

Imitation learning has become the paradigm for training robot manipulation skills. However, extending this paradigm to multi-task policy learning remains a significant challenge, which is especially pronounced in dual-arm manipulation. Compared with single-arm manipulation, dual-arm systems must handle two fundamentally different coordination modes: synchronous operation, in which both arms require precise coordination and simultaneous motion to complete a task, and asynchronous operation, in which both arms execute tasks independently in temporal sequence. These two distinct operation modes give rise to heterogeneous and diverse underlying action distributions [1,2].

In recent work, a common strategy for multi-task learning is to adopt a single policy network, namely Visual-Language-Action (VLA) models [3,4]. These methods leverage scaling laws to improve cross-task generalization through large-scale data and parameter scaling, enabling interpolation or compositional execution across multiple tasks. However, shared network parameters must simultaneously accommodate different motion modes, which leads to negative transfer across cross-mode tasks [5].

To overcome these limitations, modular policy architectures based on mixture-of-experts (MoE) models [6,7] have become an active research direction. By decomposing policies into specialized components, multi-task learning capability can be improved [8,9]. However, existing modular methods rely on observation-level or noise-level routing, producing expert modules with ambiguous or overlapping roles [10], and fail to explicitly explore different modes of dual-arm manipulation.

To address this problem, this paper proposes the LGDM framework. The architecture takes language instructions as its core. Based on shared language embeddings, it designs two parallel branches—language mode routing and visual perception—to guide the dual-mode execution layer in generating actions. Specifically, it achieves the following:

1. Language mode routing layer: It uses the temporal semantics in language instructions to determine the task mode, guiding the selection of synchronous or asynchronous policy branches in the execution layer, thereby providing mode-oriented guidance for policy learning.
2. Vision perception layer: It performs FiLM-based visual conditional perception. Through feature-wise linear modulation, it directly generates dynamic visual modulation conditions from language instructions and injects them into the visual encoder, aligning visual features with task semantics and providing adaptive visual inputs for subsequent feature processing in the policy execution branches.
3. Dual-mode execution layer: It introduces a synchronous–asynchronous dual-mode structure. To account for the differences between the two coordination paradigms in temporal dependencies and action distributions, it employs two parallel policy representation branches, each corresponding to the mapping of a different mode, and generates actions under the guidance of visually modulated features from the vision perception layer.

This architecture is inspired by the control logic of the human central nervous system over bimanual movement: the cerebral cortex first determines the cooperative intent (synchronous or asynchronous), while spinal regulatory centers dynamically coordinate specific muscle groups (neural circuits) to execute actions based on real-time somatosensory feedback [11,12].

The main contributions of this paper are as follows:

1. We propose the LGDM framework, which parallelly organizes language mode routing and visual perception modulation based on language instructions to guide action generation in the dual-mode execution layer, accomplishing mode discrimination and conditional feature modulation.
2. We adopt synchronous and asynchronous dual-mode policy branches to achieve explicit isolation of cross-mode action distributions, alleviating negative transfer in multi-task learning.
3. We conduct systematic validation on the RoboTwin 2 [13] platform; LGDM improves multi-task success rates by 14.5 percentage points over baselines and demonstrates robustness to unseen temporal-coordination instruction variants.

## 2. Related Work

### 2.1. Language-Conditioned Robotic Manipulation

Due to its broad applicability in human–robot interaction, language-conditioned robot manipulation has become a key research direction in robotics [14,15,16]. Existing methods can be broadly categorized into two classes: one leverages language models for task planning, and the other focuses on vision–language interaction for visual representation and policy learning. For task planning, Driess et al. [17] use Large Language Models (LLMs) to plan tasks and pass instructions to low-level action policies to generate robot actions. Ha et al. [18] use LLMs to scale data generation and learn language-conditioned policies with diffusion-based policy learning. For vision–language interaction, RT-1 [19] encodes multimodal tokens with a pretrained FiLM EfficientNet model and feeds them into a Transformer for multimodal information aggregation. Its successor, RT-2 [20], leverages the autoregressive generation capability of LLMs, projects visual tokens into the language space, and directly uses LLMs to generate actions. CLIPort [21] and PerAct [22] learn language-conditioned manipulation by using CLIP to connect visual observations and language instructions. Hiveformer [23] proposes a unified transformer model that jointly models language, multiple views, and history. However, most existing language-conditioned methods treat language only as a high-level planning trigger or as passive multimodal fusion features, and fail to deeply exploit the underlying physical coordination semantics in language instructions. To address this limitation, this paper distinguishes two roles of language in language-conditioned manipulation: on the one hand, based on the FiLM mechanism, language serves as passive task conditioning to modulate visual representations; on the other hand, starting from the temporal coordination semantics in instructions, language acts as an active mode signal to discriminate between synchronous and asynchronous coordination modes.

### 2.2. Multi-Task Policy Learning

Dual-arm collaborative manipulation is an important research direction in robotic manipulation, widely involved in tasks such as assembly, transport, and tool handover. Traditional dual-arm collaborative control methods mostly rely on kinematic modeling, trajectory planning, and fixed control rules, achieving collaboration by explicitly modeling geometric constraints [24,25]. Such methods depend heavily on prior engineering knowledge and are typically designed for a single task, known targets, and fixed environments; when facing a new task, they require re-modeling and re-tuning, and their adaptability to environmental disturbances and task variations is limited. In recent years, with the development of teleoperation platforms and imitation learning methods, the robot learning paradigm enables policies to learn the mapping from multimodal observations to continuous action sequences from expert demonstrations, providing a feasible technical path for acquiring complex manipulation skills [26,27]. However, such research mostly focuses on specific tasks, and determining how to extend it to open-language-instruction-driven multi-task scenarios remains a key problem.

In the field of general-purpose robotic multi-task learning, Visual-Language-Action (VLA) large models, leveraging the advantages of data and model scaling, have become the mainstream solution for improving cross-task generalization, with representative works including RDT [28] and OpenVLA [29]. These methods adopt a shared single policy network, offering implementation simplicity and strong knowledge reuse as task scale expands. However, when action distributions differ substantially across tasks, shared representations struggle to fit different motion modes simultaneously, easily leading to negative transfer across tasks. To alleviate the fitting bottleneck of monolithic policies on complex action distributions, recent work has turned toward modular policy exploration. FDP [30] represents a policy as a combination of multiple complete diffusion sub-policies and performs continuous weighted aggregation of sub-policy outputs via observation-conditioned weights, achieving action factorization at the policy level, but without structured partitioning of the policy space—mode specialization relies mainly on data-driven learning. SDP [31] embeds sparse expert layers in a Transformer diffusion backbone, selectively activating sub-networks by task to reduce active parameters and support skill reuse. MoDE [32] further uses denoising noise level as a routing condition, allocating experts along the diffusion timestep dimension to improve the efficiency of large-scale multi-task training. Variational distillation methods such as VDD [33] employ reusable denoising experts from the perspective of skill orthogonality. MoE-based methods mostly perform sparse mixing within internal sub-layers or denoising blocks; in multi-task learning research, they lack explicit physical–semantic guidance, relying on observations or noise level for expert activation, which leads to overlapping expert roles and poor interpretability. In addition, hierarchical policy learning, task decomposition, and long-horizon manipulation methods typically decompose complex tasks into high-level subgoal planning and low-level action execution, improving policy performance through skill selection, subtask sequencing, or intermediate goal generation [34,35,36]. Such methods can improve the structural organization of complex task execution, but they usually focus on task-stage division or skill reuse, requiring explicit subtask labels, predefined skill libraries, or additional stage-level supervision. For dual-arm manipulation, they focus more on what the task should do “first and then,” while rarely explicitly modeling whether the two arms should “coordinate synchronously or execute asynchronously” during execution.

Unlike the above approaches, this paper leverages the structurally meaningful prior of dual-arm synchronous and asynchronous coordination, using language-guided mode routing to select synchronous or asynchronous policy backbones at the execution layer, thereby mitigating negative transfer across modes and improving the interpretability of mode selection.

## 3. Method

**Figure 1:**
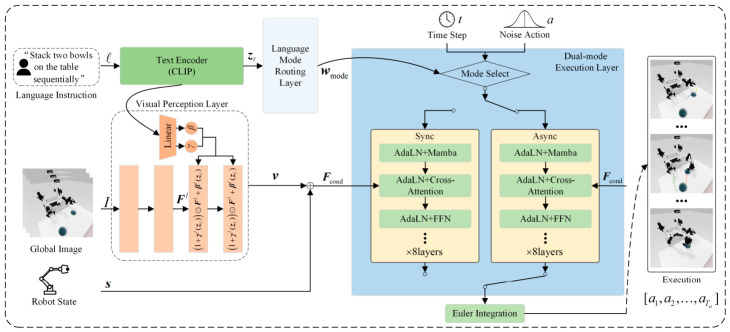
LGDM architecture.

Figure 1 illustrates the overall architecture of LGDM. The CLIP encoder produces unified semantic embeddings, which are fed into the language mode routing layer and the vision perception layer, respectively, to further dispatch the dual-mode execution layer—the three modules working in concert.

### 3.1. Problem Definition and Framework Overview

Due to differences in coordination modes, dual-arm robot manipulation tasks form two subsets: synchronous tasks $T^{s}$ and asynchronous tasks $T^{a}$. The core challenge of learning under a unified framework is that shared policy representations cannot effectively accommodate the differences in temporal characteristics and action distributions between synchronous and asynchronous modes.

Let the observation $o_{t}=(I_{t},s_{t})$ consist of an RGB image $I_{t}\in R^{3\times H\times W}$ and a proprioceptive state $s_{t}\in R^{14}$, where l is the language instruction describing the current task. The policy $\pi_{\theta }(a|o_{t-T_{o}:t},l)$ is conditioned on a historical observation window of $T_{o}$ steps, outputting an action sequence $a\in R^{T\times 14}$ of $T_{a}$ steps.

Inspired by the flow-matching policy [37], this paper models action generation as a conditional flow-matching generation process. Given the noisy action $a^{t}=(1-t)a^{0}+t\epsilon$, where $a_{0}$ is the clean action and ϵ~N(0,I), the velocity field network $v_{\theta }$ takes the joint condition $c=(o_{t},z_{l},t)$ as input, where $z_{l}$ is the text feature extracted from the instruction. The training objective is:

(1) $$L_{\mathrm{diff}}=E_{t,a^{0},\epsilon }{\left\|v_{\theta }(a^{t},t,c)-(\epsilon -a^{0})\right\|}^{2}$$

### 3.2. Language Mode Routing Layer

A frozen CLIP text encoder is used to map the language instruction to a semantic embedding:

(2) $$z_{l}=f_{\mathrm{CLIP}}(l)\in R^{d_{z}}$$

where $d_{z}$ is the dimension of the language embedding.

The routing parses the task mode from the language semantics to generate two-dimensional routing weights:

(3) $$w_{\mathrm{mode}}=\mathrm{softmax}(W_{r}z_{l}+b_{r})\in R^{2},\;W_{r}\in R^{2\times d_{z}}$$

where $w_{\mathrm{mode}}=[w_{s},w_{a}]$ represents the weights of the synchronous and asynchronous experts respectively. The routing is supervised by a cross-entropy loss:

(4) $$L_{\mathrm{router}}=\mathrm{CrossEntropy}(W_{r}z_{l}+b_{r},y)$$

where y∈{0,1} is a fixed task-level label determined by the required coordination mode of the task. y=0 denotes the synchronous mode, requiring the dual arms to move simultaneously in a coupled manner, and y=1 denotes the asynchronous mode, requiring the dual arms to execute tasks sequentially and independently according to the time sequence.

### 3.3. Visual Perception Layer

FiLM [38] modulation is inserted into the latter half of the ResNet-18 backbone network to modulate the image feature maps using the CLIP language embedding $z_{l}$. A multi-layer perceptron (MLP) network takes the task condition $z_{l}$ as input to generate the scaling γ and shifting β parameters for FiLM. For the feature map $F^{l}\in R^{C\times H^{\prime }\times W^{\prime }}$ at the *l*-th layer of ResNet, the FiLM modulation is defined as:

(5) $${\tilde{F}}^{l}=\left(1+\gamma^{l}(z_{l})\right)⊙F^{l}+\beta^{l}(z_{l})$$

After cumulative modulation at layer 3 and layer 4, followed by global average pooling and flattening, the visual feature sequence is obtained as follows:

(6) $$v=\mathrm{FiLM}\left(\mathrm{ResNet}18\left(I\right),z_{l}\right)\in R^{d_{v}}$$

After concatenating with the proprioceptive state, we obtain $F_{\mathrm{cond}}=[v,s]\in R^{d_{v}+14}$ as the key-value condition for cross-attention.

### 3.4. Dual-Mode Execution Layer

The network adopts a dual-mode structure consisting of a synchronous branch $\theta_{s}$ and an asynchronous branch $\theta_{a}$, which are two structurally identical yet parameter-independent backbones responsible for synchronous and asynchronous tasks, respectively. Given the input action feature a, it is first mapped to a hidden feature h via a linear projection. AdaLN [39] is then used to condition on the timestep, followed by sequence modeling with Mamba [40]. A cross-modal attention mechanism introduces the visual condition $F_{\mathrm{cond}}$ modulated by FiLM. Finally, the features are fed into the FFN for transformation. The structure of a single backbone is defined as:

(7) $$h^{\left(1\right)}=h+\mathrm{Mamba}\left(\mathrm{AdaL}N_{1}(h,t)\right)$$

(8) $$h^{\left(2\right)}=h^{\left(1\right)}+\mathrm{CrossAttn}\left(\mathrm{AdaL}N_{2}(h^{\left(1\right)},t),F_{\mathrm{cond}}\right)$$

(9) $$h^{\prime }=h^{\left(2\right)}+\mathrm{FFN}(\mathrm{AdaL}N_{3}(h^{\left(2\right)},t))$$

After 8 layers of processing, the final hidden state $h^{\prime }$ of each backbone is projected through its own independent output layer to predict the target velocity field for the corresponding mode: $v_{\theta_{m}}=\mathrm{Linear}(h^{\prime })$. Let $c=F_{\mathrm{cond}}$. The model variable is defined as m∈{0,1}, where $0\to \theta_{s}$, $1\to \theta_{a}$. The velocity field output is given by:

(10) $$v_{\theta }(a,t,c,m)=\left\{\begin{array}{c} v_{\theta_{s}}(a,t,F_{\mathrm{cond}}),m=0\\ v_{\theta_{a}}(a,t,F_{\mathrm{cond}}),m=1 \end{array}\right.$$

According to Equations (1) and (4), the total loss of LGDM is a weighted combination of the flow-matching generation loss and the routing supervision loss:

(11) $$L_{\mathrm{total}}=L_{\mathrm{diff}}+\lambda_{r}\cdot L_{\mathrm{router}}$$

where $L_{\mathrm{router}}$ is the routing supervision loss, trained using the ground-truth mode labels of the tasks.

During inference, given a new language instruction, the router first determines the task mode index $m=\mathrm{argmax}(w_{\mathrm{mode}})$, then activates only the corresponding motion mode. The Euler integration method is used for inference:

(12) $$a^{t-\Delta t}=a^{t}-\Delta t\cdot v_{\theta_{m}}(a,t,F_{\mathrm{cond}})$$

Training and Inference. During training, each sample is routed to the synchronous backbone $\theta_{s}$ or the asynchronous backbone $\theta_{a}$ according to its ground-truth mode label y, and the selected backbone predicts the corresponding velocity field. In parallel, the router is supervised by the cross-entropy loss in Equation (4), and the whole model is optimized with the combined objective in Equation (11) with the routing weight $\lambda_{r}=0.1$. As the language embedding $z_{l}$ is produced by a frozen CLIP encoder and the branch is selected by the discrete label y, the routing loss updates only the router, while the flow-matching loss updates the selected backbone and the FiLM visual encoder. At inference, the router performs hard routing by selecting the mode $m=\mathrm{argmax}(w_{\mathrm{mode}})$ (Equation (10) and activates only the corresponding backbone to generate actions through the Euler integration in Equation (12).

## 4. Experiment

### 4.1. Experimental Setup

**Figure 2:**
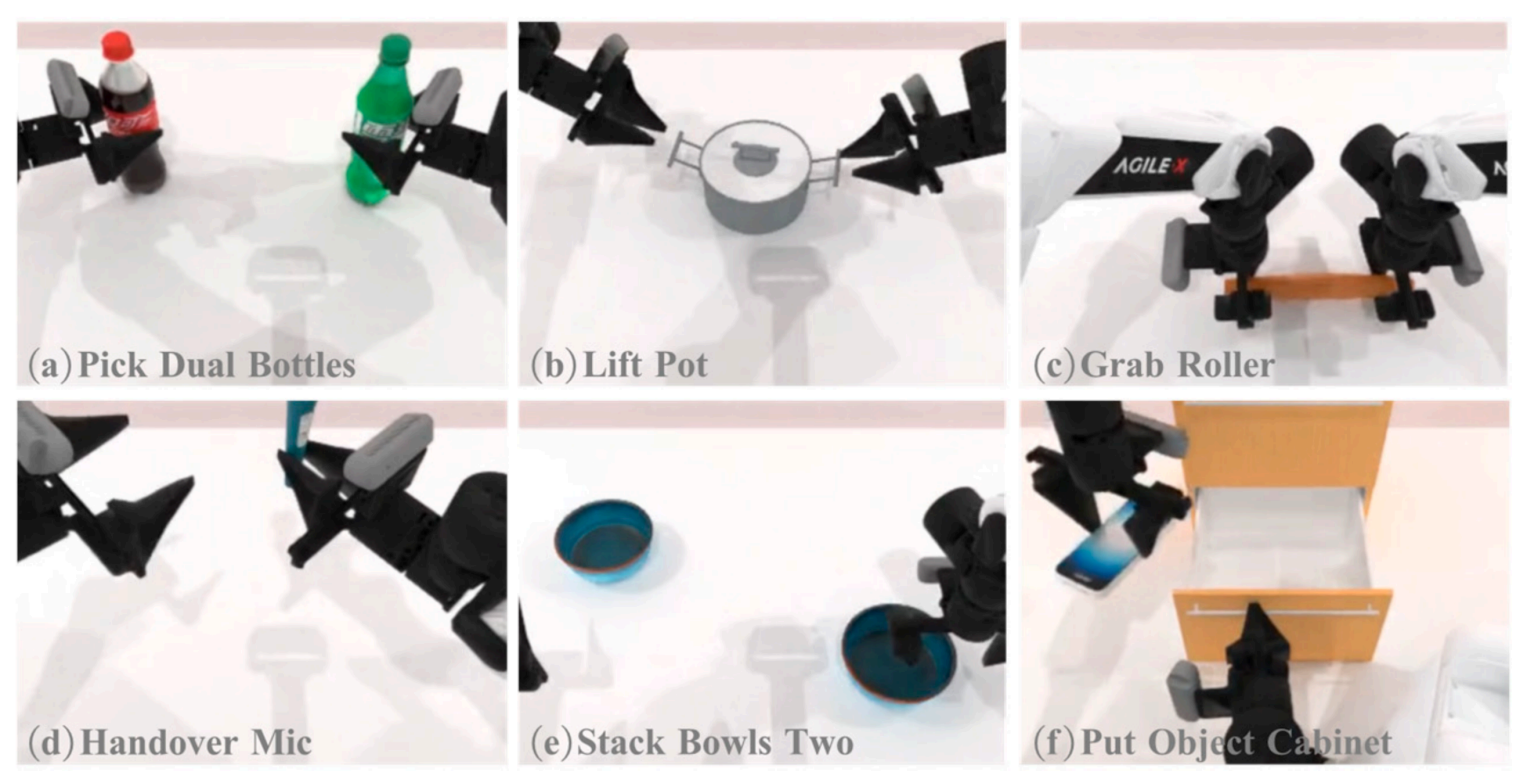
Task setup.

Platform and Tasks: Experiments are conducted on the RoboTwin 2 dual-arm simulation platform, which is equipped with two 6-DoF robotic arms. As shown in Figure 2, we select six representative dual-arm tasks for evaluation and divide them into two categories according to operation modes. The synchronous tasks include: (a) Pick Dual Bottles, where the two arms simultaneously grasp two bottles; (b) Lift Pot, where the two arms simultaneously lift a pot; and (c) Grab Roller, where the two arms cooperatively grasp and lift a cylinder. The asynchronous tasks include: (d) Handover Mic, where a microphone grasped by one robotic arm is handed over to the other arm; (e) Stack Bowls Two, where the two arms sequentially operate to stack two bowls on the table; and (f) Put Object Cabinet, where one arm first opens the cabinet door, and then the other arm places the object into the cabinet.

Dataset: For each task, 50 demonstration trajectories are collected, resulting in a total of 300 trajectories for training. Each trajectory contains language instructions, RGB images, joint states, and action sequences. Synchronous tasks are labeled as y = 0, while asynchronous tasks are labeled as y = 1.

Baseline Methods: 1. SDP: a sparse diffusion policy based on observation-driven routing; 2. MoDE: a mixture-of-experts diffusion policy based on noise-level routing; 3. RDT: a dual-arm foundation model based on diffusion transformer and supporting tri-modal fusion of vision, language, and action.

Evaluation Metric: Task Success Rate (SR) is adopted as the primary evaluation metric. In the simulation experiments, each task is evaluated 100 times under each random seed. For the main SOTA comparison experiments (SDP, MoDE, RDT, and LGDM), all algorithms are independently trained and evaluated with three random seeds $\left\{0,\;1,\;2\right\}$, and the results are reported as the mean ± standard deviation over the three runs. Meanwhile, the 95% confidence interval (95% CI) is calculated based on the number of successful and failed trials for each method, and a two-sample-proportion *Z*-test is conducted between LGDM and the strongest baseline RDT to verify the statistical significance of the performance improvement. For the ablation experiments, since they are mainly used to analyze module contributions and performance trends, we adopt a fixed random seed of 0 for rapid evaluation.

Implementation Details: The backbone network consists of eight layers with eight attention heads. The visual encoder uses ResNet-18, and the text encoder uses CLIP, which is frozen during training. The image resolution is set to 320 × 240 for both training and inference, without additional data augmentation. We use the AdamW optimizer with a batch size of 128 and a learning rate of 10^−4^, together with a cosine learning-rate schedule. The model is trained for a total of 900 epochs. The observation history length is $T_{o}$ = 3, and the model predicts an action sequence of length $T_{a}$ = 8, of which the first six steps are executed at each inference, after executing these steps, the model re-observes the environment and predicts again. The physics simulation timestep is 0.004 s, and observations/actions are sampled and executed every 15 simulation steps, yielding an effective policy control frequency of about 16.7 Hz. The number of flow-matching inference steps is set to *K* = 10, and the routing loss weight is $\lambda_{r}$ = 0.1 to stably supervise the task-level binary router while preventing the routing loss from dominating the overall objective. All experiments are conducted on a single NVIDIA RTX 3090 GPU (24 GB), with training taking approximately 60 h.

### 4.2. Comparison with Previous SOTA Architectures

To validate the overall performance advantage of LGDM, we select three existing SOTA methods as baselines, including SDP, MoDE, and RDT, and compare their performance on the mixed task set. The experimental results are shown in Table 1.

As shown in Table 1, LGDM outperforms all baseline methods across all metrics: it achieves an average success rate of 79.6%, corresponding to an absolute improvement of 14.5 percentage points over the best baseline RDT and 26.9 percentage points over the conventional SDP method. Furthermore, a two-sample-proportion *Z*-test is conducted between LGDM and RDT, yielding *Z* ≈ 9.73 and *p* < 0.001, which demonstrates that the performance improvement is statistically significant. Notably, LGDM performs slightly better on synchronous tasks (81.3%) than on asynchronous tasks (77.8%); in contrast, RDT performs slightly better on asynchronous tasks (67.2%) than on synchronous tasks (62.9%). This performance gap reveals the architectural preference of different methods for the two task types. LGDM’s language-guided dual-mode structure successfully decouples different task logics, thereby improving performance on both synchronous and asynchronous tasks.

To further analyze LGDM’s performance on individual tasks, Table 2 presents detailed success rate comparisons across the six tasks.

As shown in Table 2, LGDM achieves the best performance across all individual tasks. In particular, on the synchronous task Grab Roller, it reaches a success rate of 99.3%, improving by 26.0 percentage points over RDT; on the asynchronous task Handover Mic, it reaches 95.0%, improving by 3.3 percentage points over RDT. These results indicate that the LGDM architecture can adapt to the characteristics of both synchronous and asynchronous tasks, effectively improving task performance.

### 4.3. Ablation Experiments

To validate the necessity of core components within the LGDM framework, we conduct a series of ablation experiments, where a single random seed of 0 is used.

#### 4.3.1. Analysis of Contributions of Each Module

We select the three core modules of the LGDM framework—the language mode routing layer, the vision perception layer, and the dual-mode execution layer—and design eight fully combinatorial ablation experiments to validate the independent contributions and synergistic effects of each module. The experimental results are shown in Table 3.

Single-module analysis:

When only the language mode routing layer is used (No. 2), the overall SR improves by 2.3 percentage points. Since the policy body still uses a shared single backbone under this setting, and the routing output has not yet participated in action-generation path selection, its direct impact on final control performance is limited. This result indicates that the primary role of language mode routing is to provide synchronous and asynchronous mode-selection signals; only when combined with the dual-mode execution layer can the temporal semantics in language be further translated into effective advantages for action generation.

When only the vision perception layer is used (No. 3), the performance improves by 25.6 percentage points, indicating that language-conditioned visual encoding is an important perceptual foundation of the entire framework.

When only the dual-mode execution layer is used (No. 4), the performance improves by 20.5 percentage points, validating the effectiveness of the dual-mode structure.

Dual-module analysis:

The combination of the language mode routing layer and the vision perception layer (No. 5) yields a further 4.0-percentage-point improvement over using the vision perception layer alone (64.8%), suggesting that even without the dual-mode structure, the mode-discrimination mechanism introduced by the language mode routing layer still provides certain temporal semantics to the globally shared network, thereby bringing modest performance gains.

The combination of the language mode routing layer and the dual-mode execution layer (No. 6) improves by 25.0 percentage points over using the language mode routing layer alone (41.5%), indicating that the dual-mode structure can map routing decisions.

The combination of the vision perception layer and the dual-mode execution layer (No. 7) improves by 13.8 percentage points over using the execution layer alone (59.7%), demonstrating that visually modulated features from the vision perception layer adapt to the representation requirements of different modes.

Three-module collaboration:

Three-module synergy: When all three modules work together (No. 8), optimal performance is achieved, with a further improvement of 6.2–13.2 percentage points over any dual-module combination. This indicates that the three modules achieve strong synergistic effects: the vision perception layer injects language semantics into the visual encoder to enhance perception; the language mode routing layer transforms language semantics into mode selection; and the dual-mode execution layer maps mode selection to action-generation paths.

In addition to verifying the effectiveness of each module, Table 3 also provides evidence for the existence of cross-mode interference from the perspective of ablation experiments. When the dual-mode execution layer is not introduced, synchronous and asynchronous tasks need to share the same action-generation backbone. In No. 1, the model does not contain any of the proposed modules, and the average success rate is only 39.2%. In No. 5, although both the language mode routing layer and the vision perception layer are introduced, the execution layer still adopts a shared backbone, and the average success rate is 68.8%. In contrast, while keeping the language mode routing layer and the vision perception layer unchanged, No. 8 further introduces the dual-mode execution layer, and the average success rate increases to 79.7%, which is 10.9 percentage points higher than that of No. 5. The above results show that synchronous and asynchronous tasks have different temporal coordination patterns and action distributions. When the two are forcibly mapped to a shared action-generation backbone, cross-mode interference is likely to occur, while the dual-mode execution layer helps alleviate such interference by providing relatively independent action-generation paths for the two types of tasks.

#### 4.3.2. Analysis of Language Mode Routing

To validate the necessity of language mode routing, we design the following three routing strategies for comparison: language mode routing, which takes CLIP language embeddings as input and predicts the task mode via a router; observation-driven routing, which predicts the mode from current visual observation features, replacing the language signal; and random routing, which mixes synchronous and asynchronous mode outputs at inference time. We analyze their impact on task performance; the results are shown in Table 4.

The experimental results show that language mode routing outperforms the other routing strategies across all metrics, achieving an overall SR improvement of 9.0 percentage points over observation-driven routing and 19.7 percentage points over random routing. This indicates that language mode routing can effectively capture temporal-semantic keywords in language instructions and provide effective discriminative signals for synchronous/asynchronous mode selection. Observation-driven routing determines the corresponding mode activation from current image features, but visual features are weaker than language semantics for discriminating motion modes—temporal vocabulary in language instructions serves as a more direct discriminative signal for mode selection. Random routing (60.0%) mixes synchronous and asynchronous dual-mode outputs, breaking the parameter-path isolation of the dual-mode structure and failing to dynamically select the corresponding mode-specific policy backbone according to task semantics, making it difficult to eliminate cross-mode interference.

Table 4 further shows that the choice of routing strategy affects the task success rate: the language-based routing strategy achieves the highest average success rate, indicating that language information provides a more reliable basis than visual observation or random selection when activating the appropriate policy branch.

#### 4.3.3. Ablation on FiLM Insertion Positions

To validate the FiLM-based design of the vision perception layer and determine the optimal insertion depth, this section performs ablations by varying only the FiLM insertion positions, while keeping the language mode routing layer and the dual-backbone execution layer unchanged. The results are shown in Table 5.

Without FiLM, the average success rate drops to 66.5%, lower than all FiLM-equipped configurations, indicating that language-conditioned visual modulation is critical for multi-task perception. With FiLM applied only at Layer 4, the average success rate is 72.5%. Applying FiLM at both Layer 3 and Layer 4 (Layer 3+Layer 4) achieves the best performance, with an average success rate of 79.7%, a 7.2-percentage-point improvement over Layer 4 only. Extending FiLM to Layer 2 on top of Layer 3+Layer 4 (Layer 2+Layer 3+Layer 4) reduces the average success rate to 75.2%. Layer 3+Layer 4 achieves the highest success rate on both synchronous and asynchronous tasks, and is adopted as the final FiLM insertion configuration in this paper.

After adding the FiLM module to Layer 2, the model performance decreases, suggesting that language modulation at shallow network layers interferes with the extraction of low-level visual features. The shallow layers of ResNet mainly extract cross-task general basic features, such as edges, textures, and spatial layouts, which should remain stable across different tasks. In contrast, Layer 3 and Layer 4 encode higher-level features with richer semantic information and stronger task relevance, making them more suitable for language-conditioned modulation. Therefore, deploying FiLM modules at Layer 3 and Layer 4 can better preserve basic visual representations while guiding the high-level perception process through language.

#### 4.3.4. Analysis of Language Instruction Robustness

To validate the robustness of LGDM to unseen language instructions, the synchronous task “Lift Pot” and the asynchronous task “Stack Bowls Two” are selected as representative tasks. For each task, we use the original training instruction as the seen condition and design three unseen instruction variants. These variants preserve the manipulation target and the synchronous/asynchronous coordination semantics required for mode routing, while introducing different lexical choices and sentence structures. The experimental results are shown in Table 6.

The model achieves an average success rate of 90.0% under the training instruction. Under the three unseen instruction variants, LGDM obtains average success rates of 87.5%, 88.0%, and 87.0%, respectively. Compared with the training instruction, the performance drops are 2.5, 2.0, and 3.0 percentage points, with an average degradation of only 2.5 percentage points. Specifically, Test Instruction 1 evaluates alternative temporal expressions such as “at the same time” and “one after another”; Test Instruction 2 introduces a first-then action-order structure; and Test Instruction 3 changes the sentence structure using expressions such as “in parallel” and “placing one bowl after the other.” These results indicate that LGDM maintains strong robustness to diverse temporal-coordination expressions when the instructions preserve the coordination-mode cues required for policy execution.

#### 4.3.5. Ablation Experiment on the Number of Inference Steps K

To verify the basis for setting the flow-matching inference step number to *K* = 10 and to ensure the rationality of the experimental hyperparameter setting, this study fixes all network structures, training configurations, and random seeds, and conducts ablation experiments with different numbers of inference steps. The step values are set to *K*∈{1, 2, 10, 20}, and the average success rates of synchronous tasks, asynchronous tasks, and overall tasks under different step numbers are compared. The experimental results are shown in Table 7.

As shown in Table 7, the number of flow-matching inference steps *K* affects both task success rate and inference latency. When *K* = 1, the average success rates of synchronous tasks, asynchronous tasks, and all tasks are only 51.3%, 48.7%, and 50.0%, respectively. This indicates that single-step Euler integration is insufficient to approximate the action generation trajectory, leading to unstable policy outputs. When *K* increases from 1 to 2, the overall average success rate rises substantially from 50.0% to 79.7%, with the success rates of synchronous and asynchronous tasks reaching 81.7% and 77.7%, respectively. Meanwhile, the inference time only increases from 11.1 ms to 18.1 ms, indicating that a small number of integration steps is already sufficient for stable action generation.

Further increasing *K* does not bring additional performance improvement. When *K* = 10, the overall success rate remains 79.7%, the same as that of *K* = 2, while the inference time increases to 74.0 ms. When *K* = 20, the overall success rate slightly decreases to 79.5%, and the inference time further increases to 146.4 ms. These results show that once *K* ≥ 2, LGDM is no longer sensitive to a larger number of inference steps, and its performance quickly saturates. In the main experiments, we use *K* = 10 as a conservative configuration to ensure sufficient integration stability in the flow-matching inference process and to maintain consistency with the real-world deployment setting.

### 4.4. Failure Case Analysis

**Figure 3:**
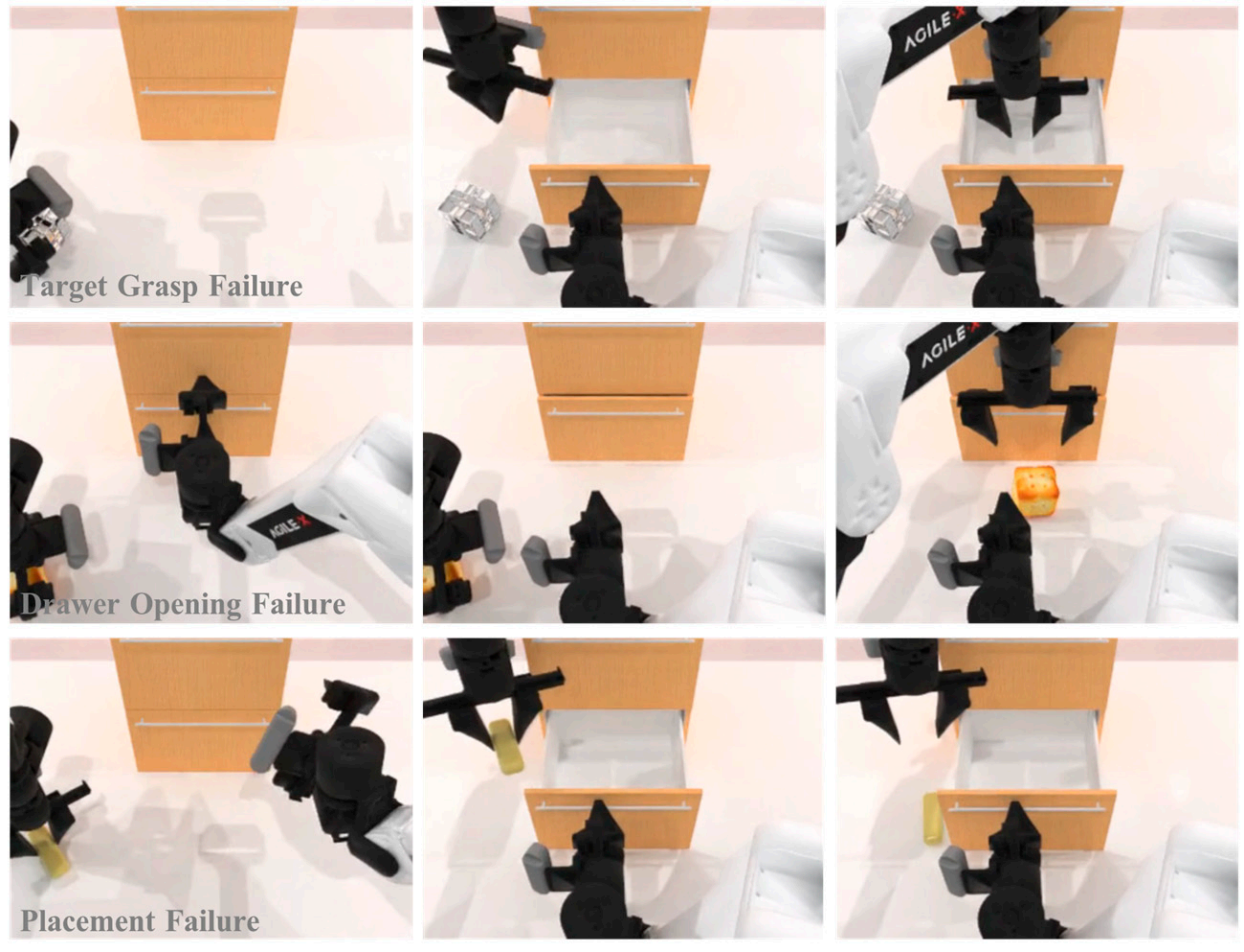
Failure cases.

The “Put Object Cabinet” task remains the most challenging task, on which the LGDM method only achieves a success rate of 50.0%. This is a multi-stage asynchronous task whose complete workflow involves actions such as grasping objects, pulling open drawers, and placing objects inside the cabinet. An error in any single step will result in the failure of the entire task. We replayed all failure trajectories and statistically analyzed the causes of failure, with the results shown in Table 8. Representative failure examples corresponding to the three main failure categories are shown in Figure 3.

Target grasp failure accounts for 70.0% of all failure cases: Ten objects with significantly different shapes are randomly selected and placed in arbitrary poses in this task. There exists the problem of inaccurate grasping positions. In addition, the gripper often fails to stably hold thin, slippery objects (such as mobile phones and playing cards).

Drawer opening failure accounts for 22.0%: The auxiliary robotic arm either fails to grasp the drawer handle successfully or cannot fully pull open the drawer, resulting in insufficient space for subsequent object placement.

Placement failure accounts for 8.0%: The object deviates slightly from the target area when released, or the gripper is not fully opened, failing to meet the criteria for task success.

## 5. Real-World Robot Results

**Figure 4:**
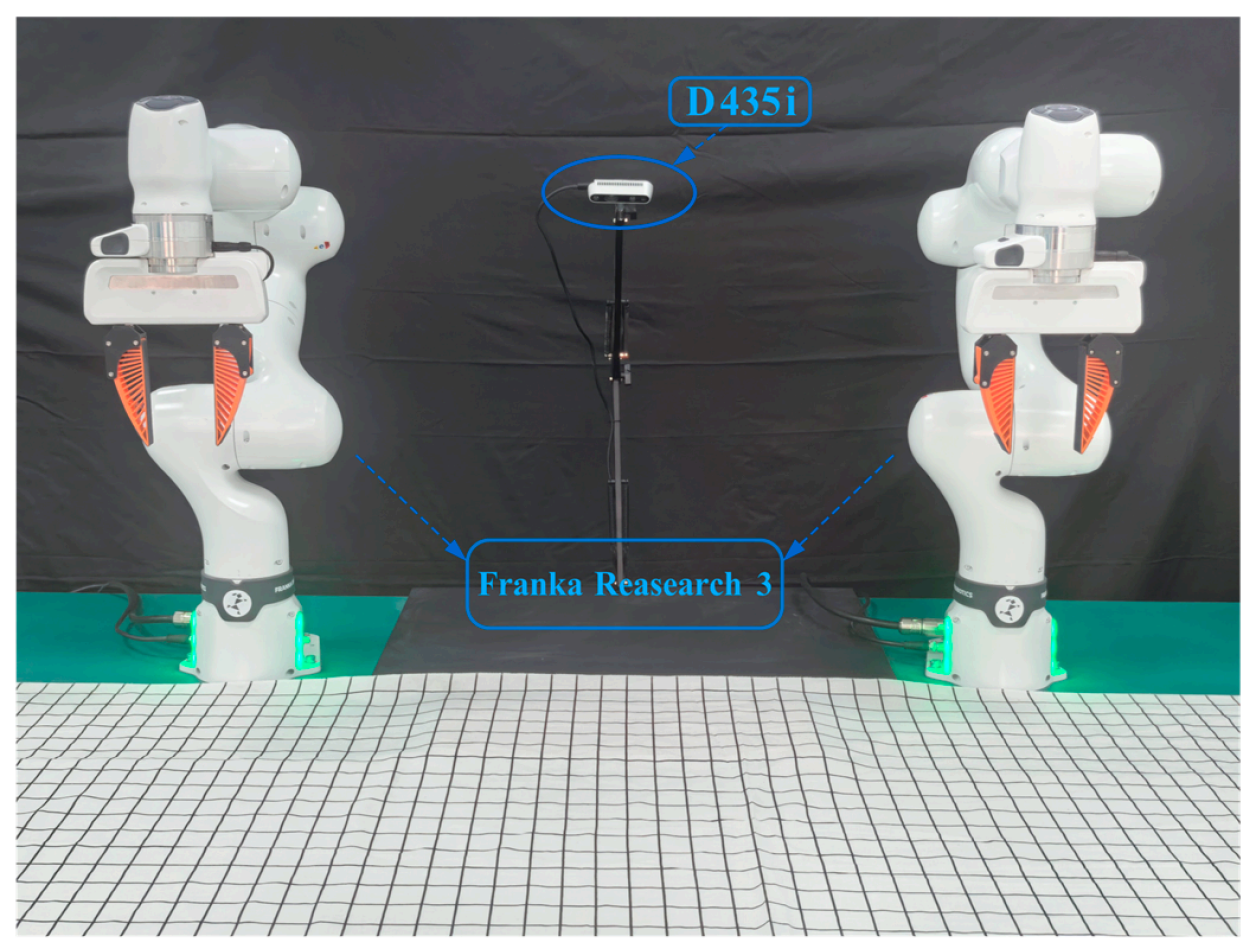
Real robot.

To further validate the performance of LGDM on a real dual-arm platform, physical validation experiments are conducted on a real dual-arm robotic system in this paper. As shown in Figure 4, the experimental platform consists of two seven-degree-of-freedom robotic arms, end-effectors, and a D435i camera. The observation inputs are consistent with those in simulation experiments, and the flow-matching inference step is fixed at K=10. The policy network runs on a workstation equipped with an Intel Core i9-14900KF CPU and an NVIDIA RTX 3090 GPU, and the control frequency of the dual-arm platform is set to 30 Hz. The real-robot models are trained directly with demonstration data collected in real-world scenarios, and the real-world dataset is independent of the simulation dataset. Data collection is implemented via primary–secondaryteleoperation, where demonstrations are performed by human experts. For each task, 50 real-world demonstration trajectories are collected for model training. The training strategy, network architecture and hyperparameters are kept consistent with those used in simulation experiments. Task success rate is adopted as the core evaluation metric. For each task, each method is evaluated over 50 independent trials. Each trial is recorded as either successful or failed according to the predefined task-specific criteria. The success rate is calculated as $\mathrm{SR}=N_{s}/N\times 100\%$, where $N_{s}$ denotes the number of successful trials and N denotes the total number of evaluation trials. In this experiment, N is set to 50 for each task. The average success rate (Avg. SR) in Table 9 is computed as the arithmetic mean of the success rates across the four tasks.

Four representative tasks are selected for validation, including two synchronous tasks, (a) Lift Pot and (b) Sort Item, and two asynchronous tasks, (c) Place Dual Shoes and (d) Handover Screwdriver. Three mainstream policy-learning algorithms, namely SDP, MoDE, and RDT, are adopted as baseline methods. All models are trained on the same real-world dataset to ensure a fair comparison. In the real-world evaluation, each method is tested under the same task settings and initial conditions. Before each trial, the two robotic arms are reset to their predefined initial poses, and the task objects are placed within the workspace according to the corresponding task configuration. No human intervention is allowed during policy execution. If grasping failure, object dropping, incorrect placement, handover failure, or failure to reach the target state occurs, the trial is regarded as a failure.

**Figure 5:**
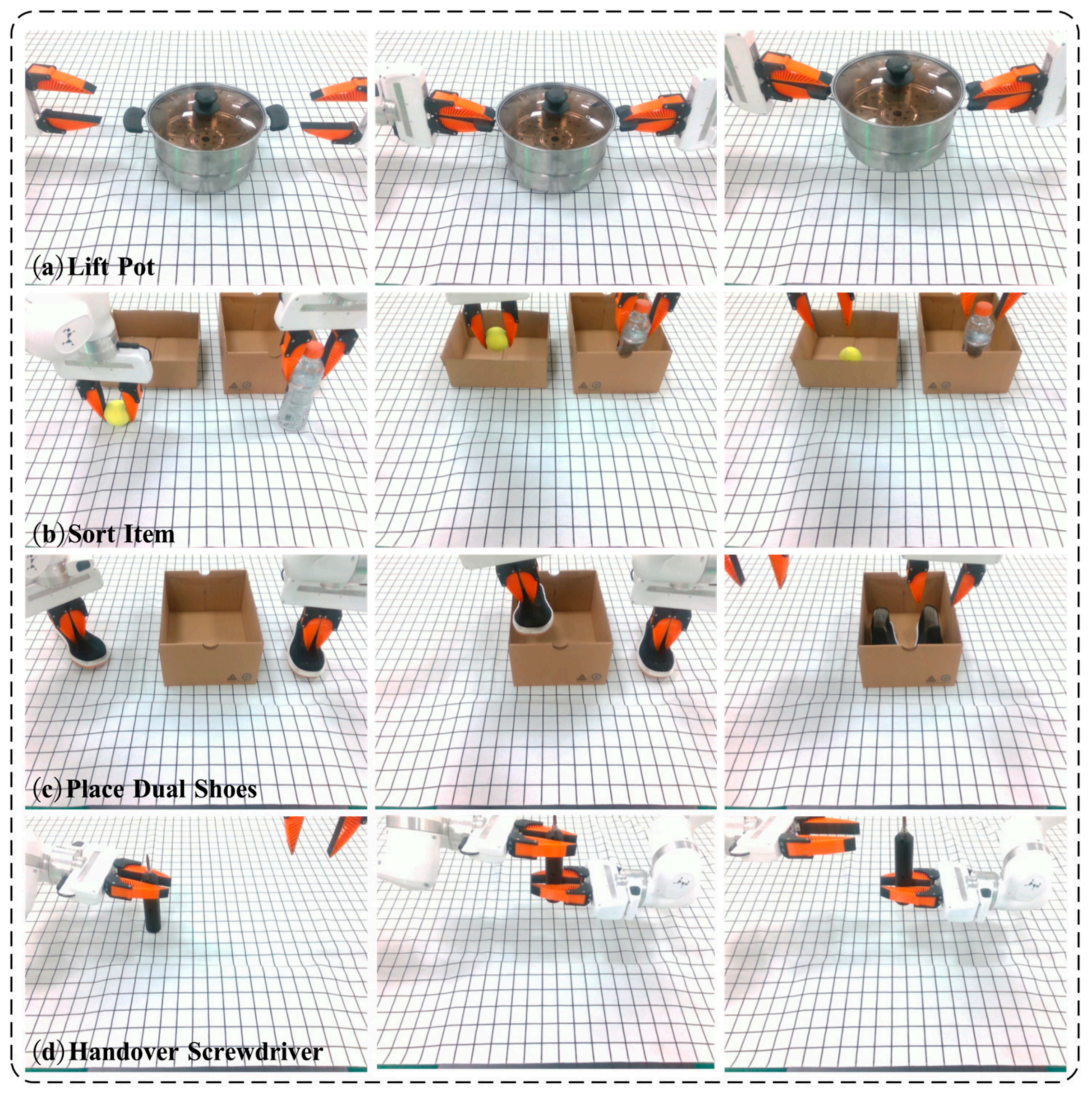
Experimental validation.

For the Lift Pot task, the two arms are required to grasp the pot from both sides and lift it cooperatively to the target height while maintaining stable dual-arm coordination. The trial is considered successful only when the pot is lifted without dropping or obvious tilting. For the Sort Item task, the two arms simultaneously grasp the target items and place them into the designated box, and success requires that all target items are correctly placed into the box. For the Place Dual Shoes task, the robot is required to place the two shoes into the shoebox sequentially, and the trial is considered successful only when both shoes are correctly placed into the shoebox without obvious overlap or misalignment. For the Handover Screwdriver task, one arm first grasps the screwdriver and moves it to the handover region, after which the other arm receives it and completes the transfer. The task is considered successful when the screwdriver is transferred between the two arms without dropping and reaches the final target state. Visual examples of policy execution in real-world scenarios are shown in Figure 5.

The quantitative results are summarized in Table 9. All results are obtained under the same real-world deployment setting with *K* = 10, where LGDM has an average policy inference latency of 76.1 ms and an end-to-end observation-to-action latency of 82.2 ms. It can be observed that LGDM achieves the best performance across all four real-world tasks, with an average success rate of 83.5%, outperforming SDP (58.5%), MoDE (55.0%), and RDT (70.0%). Furthermore, from the perspective of the 95% confidence interval (95% CI), the average success rate of LGDM has a confidence interval of [78.4%, 88.6%], which is higher than that of the strongest baseline RDT, [63.7%, 76.4%]. Meanwhile, based on a total of 200 real-robot trials across the four tasks, we conduct a two-sample-proportion Z-test between LGDM and RDT, obtaining *Z* ≈ 3.20 and *p* ≈ 0.0014. This indicates that the performance improvement of LGDM over the strongest baseline RDT is statistically significant.

In the two synchronous manipulation tasks, LGDM demonstrates significant advantages. On Lift Pot, the success rate reaches 82.0%, and on Sort Item, it further improves to 88.0%—both outperforming RDT (66.0% and 72.0%, respectively). These results indicate that language-driven synchronous mode routing can precisely dispatch the cooperative mode, maintain dual-arm motion consistency, and reliably complete grasping, lifting, and symmetric synchronous operations, validating the reliability and effectiveness of the synchronous policy mode in cooperative tasks.

In the two temporally dependent asynchronous tasks, LGDM also achieves the best performance. On Place Dual Shoes, the success rate is 78.0%, and on Handover Screwdriver, it reaches 86.0%—both higher than RDT (68.0% and 74.0%, respectively). This shows that asynchronous mode routing can accurately parse temporal semantics and drive the asynchronous policy mode to execute asymmetric action switching and cross-arm object handover in an orderly manner, demonstrating its adaptability to asynchronous operations.

## 6. Conclusions

This paper proposes the LGDM framework, which effectively addresses the synchronous and asynchronous mode discrepancy in multi-task policy learning for dual-arm robots through a language-guided dual-mode policy architecture. Using a frozen CLIP text encoder as a unified language-conditioning encoder, the language mode routing layer automatically determines the task mode from the temporal semantics in language instructions. Through the vision perception layer, visual and semantic information are aligned to enhance perceptual capability. The framework further dispatches the corresponding mode-specific policy, achieving a mapping from language mode to execution policy. Experiments on the RoboTwin 2 multi-task benchmark show that LGDM improves average success rate by 14.5 percentage points over the best multi-task baseline, and maintains a near 90% task success rate under unseen temporal-coordination instruction variants. Furthermore, in real-world physical robot deployment tests on representative tasks, the framework achieves an average success rate of 83.5%, validating its practical reliability. Ablation experiments further confirm the effectiveness of the language mode routing layer, the vision perception layer, and the dual-mode execution layer.

Although the LGDM framework proposed in this paper achieves promising experimental results on dual-arm tasks dominated by a single coordination mode, there are inherent limitations regarding its scope of applicability. The current LGDM framework identifies the coordination mode based on language instructions and keeps the corresponding synchronous or asynchronous policy branch activated throughout a single task execution. When an instruction lacks explicit coordination-mode cues or simultaneously contains synchronous and asynchronous semantic cues, the model may assign an inappropriate dominant coordination mode, causing task execution to be routed to a mismatched policy branch and leading to task failure. Accordingly, the framework is primarily applicable to dual-arm manipulation scenarios where the entire task can be characterized by one dominant coordination mode. In real-world complex operations, some dual-arm manipulation tasks exhibit multi-stage collaborative characteristics, with alternating combinations of synchronous coordination and asynchronous sequential operations throughout the task process. Constrained by the fixed mode routing mechanism, the existing model does not yet support dynamic mode recognition and adaptive switching of policy branches during task execution. Moreover, on long-horizon multi-stage tasks such as Put Object Cabinet, the performance of the framework remains limited, mainly due to the challenges of low-level contact-rich manipulation. Future research will develop a dynamically switchable multi-mode policy mechanism for mixed-mode tasks; further integrate large-scale language models to improve complex-instruction understanding and cross-task generalization performance; study the effect of demonstration data scaling and explore policy-learning methods for long-horizon contact-rich tasks; and conduct further research on inference acceleration and real-time control optimization, to accommodate diverse, highly complex dual-arm robot manipulation tasks in real-world scenarios.

## Figures and Tables

**Table 1:** Comparison of multi-task success rates.

Method	Avg. SR (Sync.)	Avg. SR (Async.)	Avg. SR	95% CI
SDP	53.4%	52.0%	52.7%	[50.39%, 55.01%]
MoDE	57.8%	52.6%	55.2%	[52.90%, 57.50%]
RDT	62.9%	67.2%	65.1%	[62.90%, 67.30%]
LGDM	81.3%	77.8%	79.6%	[77.74%, 81.46%]

**Table 2:** Comparison of individual task success rates.

Task	Mode	SDP	MoDE	RDT	LGDM
Pick Dual Bottles	Sync.	43.0 ± 2.6%	49.7 ± 3.5%	42.3 ± 2.5%	53.7 ± 2.1%
Lift Pot	Sync.	60.3 ± 1.5%	54.0 ± 2.6%	73.0 ± 1.0%	91.0 ± 3.6%
Grab Roller	Sync.	57.0 ± 2.6%	69.7 ± 1.5%	73.3 ± 3.1%	99.3 ± 1.2%
Handover Mic	Async.	64.7 ± 3.1%	53.7 ± 3.2%	91.7 ± 2.1%	95.0 ± 2.0%
Stack Bowls Two	Async.	45.0 ± 2.6%	59.3 ± 1.5%	76.7 ± 3.1%	88.3 ± 1.5%
Put Object Cabinet	Async.	46.3 ± 5.1%	44.7 ± 2.1%	33.3 ± 2.5%	50.0 ± 2.0%

**Table 3:** Ablation results of contributions from core modules.

	Language Mode Routing Layer	Perception Layer	Dual-Mode Execution Layer	Avg. SR (Sync.)	Avg. SR (Async.)	Avg. SR
1	×	×	×	47.0%	31.3%	39.2%
2	√	×	×	49.3%	33.7%	41.5%
3	×	√	×	70.3%	59.3%	64.8%
4	×	×	√	63.0%	56.3%	59.7%
5	√	√	×	76.3%	61.3%	68.8%
6	√	×	√	71.0%	62.0%	66.5%
7	×	√	√	75.7%	71.3%	73.5%
8	√	√	√	81.7%	77.7%	79.7%

**Table 4:** Ablation experiment results of routing strategies.

Routing Strategy	Avg. SR (Sync.)	Avg. SR (Async.)	Avg. SR
Language Mode Routing	81.7%	77.7%	79.7%
Observation-Driven Routing	72.0%	69.3%	70.7%
Random Routing	61.3%	58.7%	60.0%

**Table 5:** Performance under different insertion positions.

Position	Avg. SR (Sync.)	Avg. SR (Async.)	Avg. SR
No FiLM	71.0%	62.0%	66.5%
Only Layer 4	74.3%	70.7%	72.5%
Layer 3+Layer 4	81.7%	77.7%	79.7%
Layer 2+Layer 3+Layer 4	77.0%	73.3%	75.2%

**Table 6:** Robustness results for unseen language instructions.

	Lift Pot	Stack Bowls Two	Avg. SR
Training Instruction	Lift the pot with both arms simultaneously.	Stack two bowls on the table sequentially.	90.0%
Test Instruction 1	Lift the pot with both arms at the same time.	Stack two bowls on the table one after another.	87.5%
Test Instruction 2	Use the two arms together to lift the pot.	First place one bowl on the table, then stack the second bowl on top.	88.0%
Test Instruction 3	Move both arms in parallel to lift the pot at the same time.	Stack the bowls sequentially, placing one bowl after the other.	87.0%

**Table 7:** Performance and simulation policy inference time under different flow-matching inference steps.

Inference Steps *K*	Avg. SR (Sync.)	Avg. SR (Async.)	Avg. SR	Inference Time
1	51.3%	48.7%	50.0%	11.1 ms
2	81.7%	77.7%	79.7%	18.1 ms
10	81.7%	77.7%	79.7%	74.0 ms
20	81.3%	77.7%	79.5%	146.4 ms

Note: The inference time refers to the average actual runtime for a single policy prediction to generate an action sequence.

**Table 8:** Statistics of task failure causes.

Cause of Failure	Corresponding Stage	Proportion in Failures	Proportion in Total Rollouts
Target Grasp Failure	Object Grasping	70.0%	35.0%
Drawer Opening Failure	Drawer Pulling	22.0%	11.0%
Placement Failure	Cabinet Placement	8.0%	4.0%
Total	-	100.0%	50.0%

**Table 9:** Quantitative comparison of real-world robot experiments across four dual-arm manipulation tasks.

Task	Mode	SDP	MoDE	RDT	LGDM
Lift Pot	Sync.	60.0%	50.0%	66.0%	82.0%
Sort Item	Sync.	58.0%	62.0%	72.0%	88.0%
Place Dual Shoes	Async.	56.0%	52.0%	68.0%	78.0%
Handover Screwdriver	Async.	60.0%	56.0%	74.0%	86.0%
Avg. SR	-	58.5%	55.0%	70.0%	83.5%
95% CI	-	[51.7%, 65.3%]	[48.1%, 61.9%]	[63.7%, 76.4%]	[78.4%, 88.6%]

Note: Sync. and Async. denote synchronous and asynchronous tasks, respectively. Values indicate task success rates (%), and Avg. SR denotes the average success rate across the four real-world tasks. The 95% CI denotes the confidence interval of the average success rate across all real-world evaluation trials.

## Data Availability

The data presented in this study are available on request from the corresponding author.
